# Chest wall muscle area, ventilatory efficiency and exercise capacity in systemic sclerosis

**DOI:** 10.1007/s11739-024-03751-z

**Published:** 2024-09-17

**Authors:** Nicola Galea, Amalia Colalillo, Serena Paciulli, Chiara Pellicano, Martina Giannetti, Emanuele Possente, Gregorino Paone, Antonella Romaniello, Maurizio Muscaritoli, Edoardo Rosato, Antonietta Gigante

**Affiliations:** 1 Department of Radiological, Oncological and Pathological Sciences, Sapienza University of Rome, Rome, Italy; 2 Department of Translational and Precision Medicine, Sapienza University of Rome, Rome, Italy; 3 Department of Cardiovascular, Respiratory, Nephrologic, Anesthesiologic, Geriatric Sciences La Sapienza University of Rome, Rome, Italy; 4 Division of Cardiology, Sant’Andrea Hospital, Sapienza University of Rome, Rome, Italy

**Keywords:** Systemic sclerosis, Chest wall area, Interstitial lung disease, Cardiopulmonary exercise testing, Low muscularity

## Abstract

To investigate the potential contribution of chest wall muscle area (CWMA) to the ventilatory efficiency and exercise capacity in patients with Systemic Sclerosis (SSc) without interstitial lung disease (ILD). Forty-four consecutive SSc patients [*F* = 37, median age 53.5 years (IQR 43.5–58)] were examined using chest high-resolution computed tomography (HRCT), pulmonary function tests and cardiopulmonary exercise testing (CPET). The CWMA was evaluated at the level of the ninth thoracic vertebra on CT images by two independent evaluators blinded to the patient information. CPET parameters analyzed were maximum oxygen uptake (VO2 max) and VO2 at anaerobic threshold (VO_2_@AT); minute ventilation (VE); maximum tidal volume (VT). A statistically significant positive correlation was found between CWMA and maximum workload (r = 0.470, *p* < 0.01), VO2 max ml/min (*r* = 0.380, *p* < 0.01), VO2@AT (*r* = 0.343, *p* < 0.05), VE (*r* = 0.308, *p* < 0.05), VT (*r* = 0.410, *p* < 0.01) and VO2/heart rate (*r* = 0.399, *p* < 0.01). In multiple regression analysis, VO2 max (ml/min) was significantly associated with CWMA [*β* coefficient = 5.226 (95% CI 2.824, 7.628); *p* < 0.001], diffusing capacity for carbon monoxide (DLco) [*β* coefficient = 6.749 (95% CI 1.460, 12.039); *p* < 0.05] and body mass index (BMI) [*β* coefficient = 41.481 (95% CI 8.802, 74.161); *p* < 0.05]. In multiple regression analysis, maximum workload was significantly associated with CWMA [*β* coefficient = 0.490 (95% CI 0.289, 0.691); *p* < 0.001], DLco [*β* coefficient = 0.645 (95% CI 0.202, 1.088); *p* < 0.01] and BMI [*β* coefficient = 3.747 (95% CI 1.013, 6.842); *p* < 0.01]. In SSc-patients without ILD, CWMA represents an important variable in exercise capacity and can be evaluated by the mediastinal window available in the HRCT images required for lung disease staging.

## Introduction

Systemic sclerosis (SSc) is an autoimmune disease characterized by microvascular dysfunction, immune dysregulation and progressive fibrosis of the skin and internal organs causing multi-organ damage, often involving the lungs [1]. Interstitial lung disease (ILD) associated with SSc (SSc-ILD) is a common manifestation with variable clinical course, occurring in up to 50% of patients and accounting for 40% of deaths [2]. Chest high-resolution computed tomography (HRCT) and pulmonary function tests (PFTs) are used for the diagnostic assessment of SSc-ILD [2].

Declining values for forced vital capacity (FVC) and diffusing capacity for carbon monoxide (DL_CO_) are observed in the ILD progression in association with other factors such as short disease duration, male gender, skin fibrosis and high inflammatory markers [2, 3]. Recently, Nawata et al. in a small retrospective study investigated the potential contribution of accessory muscle atrophy to FVC reduction in SSc-ILD confirming that most SSc-ILD patients lose their accessory respiratory muscles during a 3-year follow-up [4].

The ventilatory efficiency, regardless of ILD, has been evaluated in SSc patients with cardio-pulmonary exercise testing (CPET) [5]. In a previous study we have demonstrated in SSc patients the reduced exercise tolerance assessed by CPET in absence of cardiac and pulmonary involvement [5]. Changes in pulmonary blood vessels occur early in asymptomatic SSc patients and abnormalities in maximum (max) rate (V) of oxygen (O₂) used during exercise (VO_2_ max) and high ventilation/carbon dioxide production (VE/VCO_2_) slope were found at CPET [5, 6].

CPET is a non-invasive, dynamic assessment providing an evaluation of respiratory, cardiovascular, metabolic and muscular response to physical effort. In clinical practice it is recommended for patients with diagnosis of pulmonary hypertension (PH) and among parameters analyzed, VO_2_ max (index of reduced exercise capacity) and high VE/VCO_2_ slope (reflects reduced ventilatory efficiency) have proved to be useful and prognostic on outcome [7].

Beyond the known factors contributing to ventilatory efficiency and exercise capacity in patients with PH, other central and peripheral factors contributing to changes in exercise tolerance, including skeletal and respiratory muscle dysfunction [8].

In fact, both respiratory and limb muscle dysfunction, often underestimated in the clinical evaluation, is increasingly as a potential contributor to several manifestations of PH [9].

With this background, the aim of the present study was to investigate the potential contribution of the chest wall muscle area (CWMA) to the ventilatory efficiency and exercise capacity in SSc patients assessed by CPET.

## Methods

This single-center, retrospective study enrolled consecutive patients with SSc without SSc-ILD with FVC > 80% and DLCO > 60%.

Patients were selected based on the fulfilment of the 2013 ACR/EULAR classification criteria for SSc [10] who had followed up with cardiopulmonary function with PFTs, CPET, HRCT and Doppler echocardiogram. Patients with heart failure with both reduced and preserved ejection fraction, primary and secondary ILD, pulmonary hypertension, valvular heart diseases, arrhythmias and conduction disorders, neuromuscular diseases, cancer, eating disorders, myositis, end stage kidney disease, smokers were excluded.

At the time of enrolment, all SSc patients were undergoing treatment with calcium channel blockers (nifedipine 30 mg/day). None of the patients was treated in the last 6 months before the enrolment with immunosuppressive agents (e.g. rituximab, mycophenolate mofetil or prednisone dose > 10 mg/day).

All patients who met inclusion and exclusion criteria were consecutively enrolled. lcSSc and dcSSc patients were enrolled regardless of disease duration. There is no selection bias because the two groups are numerically equivalent (22 lcSSc and 22 dcSSc).

This study was approved by the Ethics Committee of Sapienza University (IRB 0304) and written informed consent was obtained from all patients.

### Clinical assessment of SSc

According to classification, SSc patients were classified based on the degree of skin involvement in diffuse cutaneous (dc) or limited cutaneous (lc) SSc [11]. The modified Rodnan skin score was used for skin thickening [12], disease activity index (DAI) [13] and disease severity scale (DSS) [14] were recorded.

Nailfold videocapillaroscopy was performed with videocapillaroscope (software Pinnacle Studio Version 8) equipped with a 500 × optical probe. The patterns identified within the “SSc pattern” include early, active, and late [15].

### Study procedures

#### Pulmonary function tests

Body plethysmography parameters of flows and volumes [(FEV1, forced expiratory volume in the 1st second), FVC (Forced vital capacity), FEV1/FVC)] and single breath carbon monoxide (CO) diffusing capacity (DL_CO_), corrected for hemoglobin concentration, total lung capacity (TLC) were recorded with a Quark PFT 2 spirometer (Cosmed) and expressed according to the standards recommended by the American/European Respiratory Society [16, 17]. All spirometric parameters are expressed as percentage of predicted.

#### Cardiopulmonary exercise test

A maximal symptom-limited CPET was performed on an electronically braked cycloergometer (Ergoline-800, Mortara, Bologna, Italy), the subject wearing a nose clip and breathing through a mass flow sensor (Quark PFT, Cosmed, Rome, Italy) connected to a saliva trap. A personalized ramp exercise protocol was performed, aiming at a test duration of 10 ± 2 min [18]. The exercise was preceded by few minutes of resting breath-by-breath gas exchange monitoring and by a 3 min unloaded warm-up. The maximum (max) rate (V) of oxygen (O₂) consumption in one minute per kilogram of body weight (ml/kg/min) (VO_2_ max) was calculated according to the standard formula. The lactate threshold (LT) was identified through a V-slope analysis of VO2 and carbon dioxide production (VCO_2_), as well as of minute ventilation (VE), and it was confirmed through specific behaviour of O_2_ (VE/VO_2_) and CO_2_ (VE/VCO_2_) ventilatory equivalents and pressure end-tidal of O_2_ (PetO_2_) and CO_2_ (PetCO_2_). The end of the isocapnic buffering period was identified when VE/VCO_2_ increased, and end-tidal pressure of CO_2_ decreased. The relation between VO_2_ and workload (VO_2_/WR) was calculated as the slope of the linear relationship between VO_2_ and WR from the beginning of loaded exercise to the end of the exercise test. The relation between VE and VCO_2_ (VE/VCO_2_ slope) was calculated as the slope of the linear relationship between VE and VCO_2_ from one minute after the beginning of loaded exercise to the end of the isocapnic buffering period. CPET was self-terminated by the subjects when they claimed that they had achieved maximal effort. However, we considered maximal effort as achieved if the respiratory exchange ratio (RER), calculated as the ratio between VCO_2_ and VO_2_, was above 1.05. A 12-lead ECG, arterial systemic blood pressure and arterial O_2_ saturation (integrated pulse-oxymeter) were also recorded at baseline and during effort [19]. All CPET were executed and analyzed by two physicians blinded to patients’ clinical features.

#### High-resolution computed tomography

**Fig. 1:**
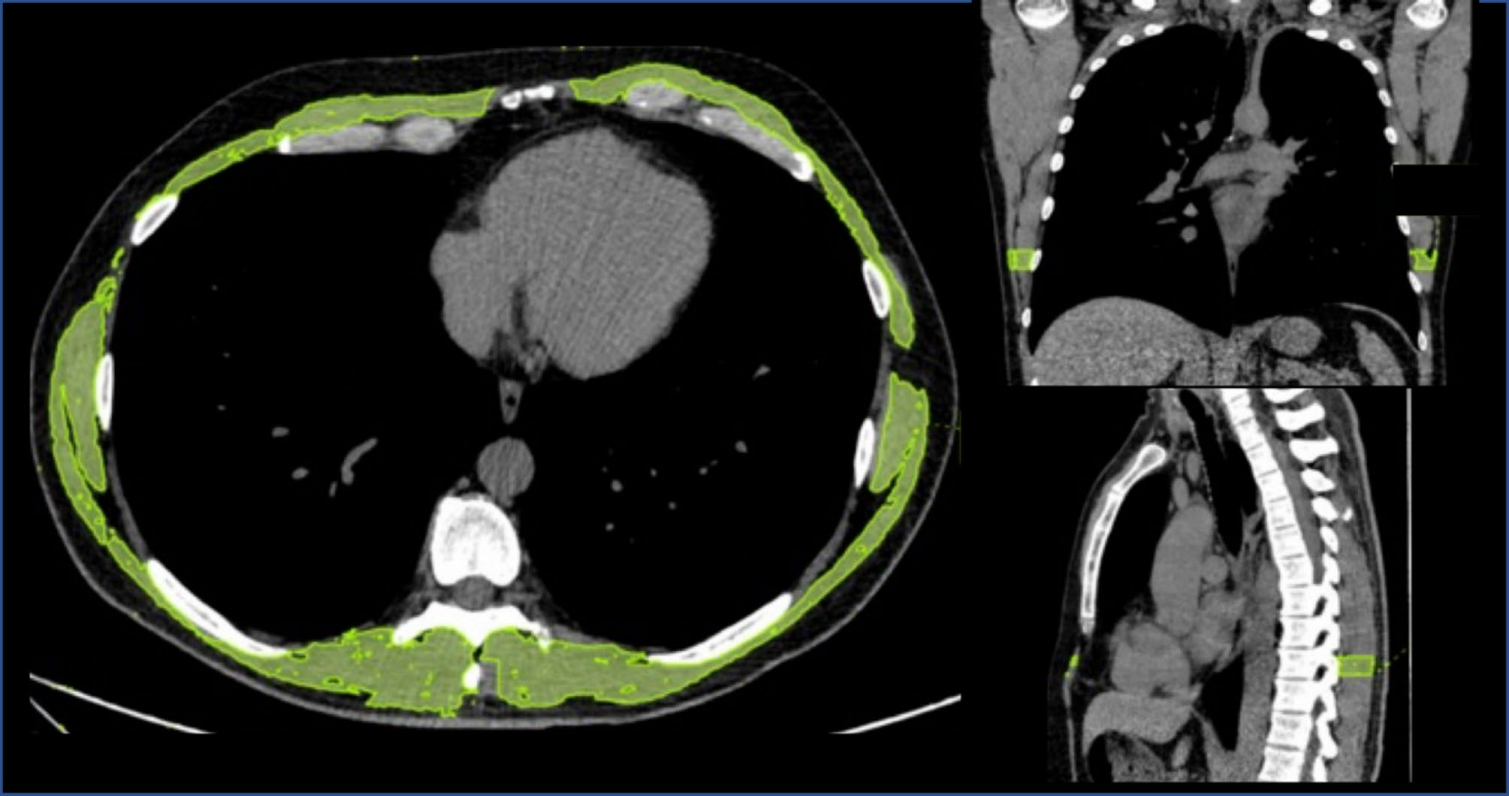
The chest wall muscle area was semi-automatically segmented on axial CT images at the level of the ninth thoracic vertebra, using the mediastinal viewing window. All soft tissues with Hounsfield values comprised between 0 and 100, located outside the rib cage (excluding ribs, vertebrae and lung parenchyma), were segmented (green color) and considered as accessory respiratory muscles (color figure online)

HRCT of the chest was performed at baseline using a 128 slice CT scanner (Ingenuity Core, Philips, Amsterdam, Netherlands) and classified according to score of ground-glass opacities, fibrosis and to exclude ILD [20]. The CWMA was evaluated at the level of the ninth thoracic vertebra on the mediastinal window of CT, because the image at this level includes accessory respiratory muscles, such as the latissimus dorsi muscle, erector spinae muscle, serratus anterior muscle, inferior trapezius muscle and inferior pectoralis major muscle, with minimal inclusion of breast tissue and rectus abdominis muscle tissue [4]. The accessory respiratory muscles play a major role in forced expiration and thus are a major contributor to FVC measurement, whereas inspiratory muscles, such as the diaphragm and intercostal muscles activate during tidal breathing [21]. The accessory respiratory muscles were semi-automatically traced (Fig. 1), using a threshold-based approach (including all tissue with density comprised between 0 and 100 Hounsfield units, outside from rib cage) and total area was quantified using Vitrea Advanced Visualization software (Canon Group, Minnetonka, MN). Breast parenchyma and costal cartilages were manually excluded. The mean of two measurements was used for analysis.

#### Doppler echocardiography

Doppler echocardiography was performed by a senior cardiologist with the General Electric Vivid S5 apparatus (GE Medical Systems, Israel Ltd.). Left ventricular ejection fraction and systolic pulmonary arterial pressure (sPAP) was determined from peak tricuspidal jet velocity using the simplified Bernoulli equation and combining this value with an estimate of the right atrium pressure:

sPAP = 4(V)2 + RA pressure, where V is the peak velocity (in meters per second) of the tricuspid valve regurgitant jet, and RA pressure is estimated from inferior vena cava diameter and respiratory changes [22, 23].

### Statistical analysis

SPSS version 26.0 software was used for statistical analysis. After evaluation of normality by Shapiro–Wilk test, continuous variables were expressed as median and IQR and categorical variables were expressed as absolute frequency and percentage. Differences between groups were evaluated by Student’s or Mann–Whitney’s *U* test. Differences between categorical variables were evaluated by the chi-square or Fisher exact test. The Pearson or Spearman correlation test was used for bivariate correlations. Multiple regression analysis was performed to evaluate relationship between dependent variables (VO_2_ max or maximum workload) and independent variables [age, body mass index (BMI), DLco, sPAP and chest wall muscle area]. *p*-values < 0.05 were considered significant.

## Results

In this study were enrolled 44 SSc patients [*F* = 37, median age 53.5 years (IQR 43.5–58)], 22 (50%) SSc patients had dcSSc and 22 (50%) lcSSc. Table 1 summarizes demographic and clinical features of SSc patients.

**Table 1:** Demographic and clinical features of SSc patients

Age, years, median (IQR)	53.5 (43.5–58)
Female, *n* (%)	37 (84.1)
BMI, Kg/m^2^, median (IQR)	22.1 (20.55–26.3)
dcSSc, *n* (%)	22 (50)
Disease duration, years, median (IQR)	9.5 (5.5–15)
mRSS, median and IQR	11 (7–16.5)
SSc-specific autoantibodies	
Anti-topoisomerase I, *n* (%)	23 (52.3)
Anti-centromere, *n* (%)	8 (18.2)
None, *n* (%)	13 (29.5)
Nailfold capillaroscopic pattern	
Early, *n* (%)	6 (13.6)
Active, *n* (%)	16 (36.4)
Late, *n* (%)	22 (50)
DAI, median (IQR)	1.5 (0.88–2.75)
DSS, median (IQR)	4 (3–6)
FVC, % of predicted value	100 (88–107)
TLC, % of predicted value	93 (81.5–103.5)
DLco, % of predicted value	77 (68.5–86)
EF, %	60 (60–62.5)
sPAP, mmHg	28 (25–30)

*SSc* systemic sclerosis, *BMI* body mass index, *dcSSc* diffuse cutaneous systemic sclerosis, *mRSS* modified rodnan skin score, *DAI* disease activity index, *DSS* disease severity scale, *FVC* forced vital capacity, *TLC* total lung capacity, *DLco* diffusing capacity of the lung for monoxide carbon, *EF* ejection fraction, *sPAP* systolic pulmonary arterial pressure, *IQR* interquartile range

Median CWMA was 43.9 cm^2^ (IQR 36.8–56.5), median VO_2_ max was 1211 ml/min (IQR 1028.5–1451) and median maximum workload was 80 W (IQR 62–105.5). Table 2 shows the CWMA and CPET parameters of SSc patients.

**Table 2:** Chest wall muscle area and cardiopulmonary exercise testing parameters of SSc patients

Chest wall muscle area, cm^2^, median (IQR)	43.9 (36.8–56.5)
RER, median (IQR)	1.21 (1.13–1.27)
Maximum workload, W, median (IQR)	80 (62–105.5)
VO_2_ max, ml/min, median (IQR)	1211 (1028.5–1451)
VO_2_ max, % of predicted value, median (IQR)	81 (72–87)
VO_2_@AT, ml/min, median (IQR)	789.5 (679–952)
VE/VCO_2_ slope, median (IQR)	29.75 (26.25–33.9)
HR rest, bpm, median (IQR)	77 (71–91)
HR max, bpm, median (IQR)	152.5 (140–163.5)
SBP/DBP rest, mmHg, median (IQR)	105 (100–120)/70 (60–80)
SBP/DBP max, mmHg, median (IQR)	160 (150–180)/90 (80–100)
SpO_2_max, %, median (IQR)	98 (97–99)

*SSc* systemic sclerosis, *RER* respiratory exchange ratio, *VO*_*2*_* max* maximum oxygen uptake; *VO*_*2*_*@AT* VO_2_ at anaerobic threshold, *VE/VCO*_*2*_* slope* minute ventilation/carbon dioxide production, *HR* heart rate, *SBP* systolic blood pressure, *DBP* diastolic blood pressure, *SpO*_*2*_ arterial oxygen saturation, *IQR* interquartile range

Chest wall muscle area was significantly higher in male patients with SSc compared to female patients with SSc [80.8 (IQR 72.8–89.8) vs 42.2 (IQR 35.7–47.4) cm^2^, *p* < 0.001]. We did not find significant differences between CWMA and other clinical variables.

A statistically significant positive correlation was found between CWMA and maximum workload (*r* = 0.470, *p* < 0.01), VO_2_ max ml/min (*r* = 0.380, *p* < 0.01), VO_2_@AT (*r* = 0.343, p < 0.05), VE (*r* = 0.308, *p* < 0.05), VT (*r* = 0.410, *p* < 0.01) and VO_2_/HR (*r* = 0.399, *p* < 0.01). Moreover, slightly significant positive correlation was found between chest wall muscle area and body mass index (BMI) (*r* = 0.255, *p* < 0.05). We did not find a statistically significant correlation between CWMA and ejection fraction (*r* = 0.080, *p* > 0.05) or FVC/DLCO (*r* = 0.112, *p* > 0.05). No significant correlations were found between CWMA and other echocardiographic, PFTs and CPET variables.

A statistically significant positive correlation was found between VO_2_ max (ml/min) and BMI (*r* = 0.364, *p* < 0.05) and DLco (*r* = 0.306, *p* < 0.05), conversely a negative correlation was found between VO_2_ max (ml/min) and sPAP (*r* = − 0.326, *p* < 0.05). No significant correlation was observed between VO_2_ max (ml/min) and FVC or other clinical variables.

A statistically significant positive correlation was found between maximum workload and BMI (*r* = 0.363, *p* < 0.01) and DLco (*r* = 0.291, *p* < 0.05), conversely a negative correlation was found between maximum workload and sPAP (*r* = − 0.326, *p* < 0.05). No significant correlation was observed between maximum workload and FVC or other clinical variables.

In multiple regression analysis, VO2 max (ml/min) was significantly associated with CWMA [*β* coefficient = 5.226 (95% CI 2.824, 7.628); *p* < 0.001], diffusing capacity for carbon monoxide (DLco) [*β* coefficient = 6.749 (95% CI 1.460, 12.039); *p* < 0.05] and body mass index (BMI) [*β* coefficient = 41.481 (95% CI 8.802, 74.161); *p* < 0.05] (Table 3).

**Table 3:** Multiple regression analysis in SSc patients

Variables	*β* coefficient (CI)	*p*
VO2 ml/min	Dependent variable	
Age	− 5.919 (− 14.706 to 2.867)	> 0.05
BMI	41.481 (8.802 to 74.161)	< 0.05
Chest wall muscle area	5.226 (2.824 to 7.628)	< 0.001
DLco	6.749 (1.460 to 12.039)	< 0.05
sPAP	− 2.396 (− 14.020 to 9.228)	> 0.05

Maximum workload	Dependent variable	
Age	− 0.704 (− 1.439 to 0.031)	> 0.05
BMI	3.747 (1.013 to 6.482)	< 0.01
Chest wall muscle area	0.490 (0.289 to 0.691)	< 0.001
DLco	0.645 (0.202 to 1.088)	< 0.01
sPAP	− 0.540 (− 1.513 to 0.432)	> 0.05

*SSc* systemic sclerosis, *BMI* body mass index, *VO*_*2*_* max* maximum oxygen uptake, *DLco* diffusing capacity of the lung for monoxide carbon, *sPAP* systolic pulmonary arterial pressure

In multiple regression analysis, maximum workload was significantly associated with CWMA [*β* coefficient = 0.490 (95% CI 0.289, 0.691); *p* < 0.001], DLco [*β* coefficient = 0.645 (95% CI 0.202, 1.088); *p* < 0.01] and BMI [*β* coefficient = 3.747 (95% CI 1.013, 6.842); *p* < 0.01] (Table 3).

## Discussion

The present study showed for the first time that in SSc patients without ILD disease, the exercise capacity and maximum workload achievement is associated with CWMA and BMI.

CPET defines maximum exercise capacity through measurement of VO_2_ max and its evaluation during a maximal symptom limited CPET is the most proven method to assess functional capacity. VO_2_ max is an important predictor of prognosis in patients with heart failure and it is influenced by other factors not related to cardiac involvement such as gender, age and muscle mass [24].

In the previous studies, VO_2_ max was the best predictor of mortality both in patients with heart failure with reduced [25] and preserved [26] ejection fraction.

CPET has been used in SSc to establish the etiology of breathlessness. From the studies, it was possible to establish that in CPET, rather than parenchymal or vascular pulmonary disease, other factors may contribute to exercise limitation [27, 28]. Cardiac function may be impaired as result of pulmonary fibrosis, as well as the myocardial tissue can be also directly affected by SSc-related immune-mediated inflammation, characterized by myocardial edema, fibrosis and microvascular dysfunction [29]. However, Ross et al. [27] confirmed that SSc patients without cardiopulmonary involvement have reduced exercise capacity with VO2 max of 70% vs 98% in healthy controls and in most of SSc patients maximum workload was below the threshold associated with functional disability. Since there was no correlation between impaired exercise capacity and left ventricular ejection fraction, the authors concluded that more sensitive measures of organ involvement, such as diffuse myocardial fibroinflammatory disease and skeletal muscle oedema, were associated with impaired exercise tolerance [27]. Also, De Oliveira et al. [28] found in SSc patients without pulmonary involvement the reduction of exercise capacity probably due to abnormal reduced skeletal muscle blood flow.

Indeed, respiratory muscles and joints may also be primarily affected by SSc, and the resulting hypotrophy of the chest wall muscles may have a role on the exercise tolerance.

VO2 max is the most important parameter that could indicate changes with oxygen transport or oxygen extraction at tissue level or in pulmonary, neuromuscular and musculoskeletal diseases [30]. Only one study in the past investigated the potential contribution of accessory respiratory muscle atrophy to the decline of FVC in patients with SSc-associated ILD [4]. The authors found, in interval 1.3 years, that CWMA decreased significantly with time while changes in FVC and total ILD extent were variable among patients. A negative correlation was found between FVC and ILD extent and positive correlation was found between FVC and change in the CWMA. At multivariate analysis, changes in total ILD extent and CWMA were independent contributors to FVC decline. Thus, the atrophy of accessory respiratory muscles seems to have a role in the impairment of lung function.

In our study, in multiple regression analysis, VO2 max (ml/min) and maximum workload were significantly associated with CWMA.

The first evidence of impaired respiratory muscle and diaphragm function was studied in patients with PH. Meyer et al. [31] observed reduced maximal inspiratory and expiratory pressures and they supposed that it could be related to generalized skeletal muscle weakness due to decreased systemic oxygen transport with subsequent muscle atrophy. The mechanisms that lead to muscle dysfunction in those patients can be related to impaired cardiac output not able to increases in oxygen demand by the skeletal muscles during exercise; chronic arterial hypoxemia, inflammation, increased sympathetic tone that reduce the perfusion of the skeletal muscles increasing peripheral vascular resistance; the inhibition of hormonal/anabolic pathways, such as the insulin-signaling pathway, sedentary lifestyle and endothelial dysfunction [9, 31].

Recently, was demonstrated that low cross-sectional area of the erector spinae muscles is a risk factor for all cause mortality in idiopathic pulmonary fibrosis (IPF) patients [32] and low thoracic muscle mass index obtained from the preoperative thoracic CT is an independent predictor of postoperative adverse outcomes in patients following lobectomy via thoracotomy for lung cancer [33]. Although in both cited studies the sections chosen for the evaluation of muscle mass are different from the one chosen in our study (at the level of the 12th [32] or 5th [33] vs 9th thoracic vertebra), both seem to confirm the results of our study demonstrating that the evaluation of muscle trophism can be of help in the evaluation and management of restrictive lung diseases.

To the best of our knowledge, in literature, there is a lack of studies between CWMA in autoimmune disease and in particular SSc, although in SSc, low muscularity is frequently encountered, ranging 20–22% [34]. Malnutrition, gastrointestinal involvement, steroid therapy, endothelial dysfunctions, microvascular changes, abnormal angiogenesis and physical inactivity contribute to the development of low muscularity among SSc patients that negatively impacts on morbidity, mortality, rehospitalization rates, quality of life and healthcare costs [35–37]. There is therefore an urgently needing for an early assessment of thoracic muscle damage and muscle trophism in relation to SSc-related complications.

The evaluation of muscle trophism in SSc patients with imaging modalities is challenging, since a single morphological or structural parameter may only partially reflect the effective degree of muscle tonicity. In particular, a limitation of our study is that we did not evaluate the muscle inflammatory involvement or metabolism, which can be identified by magnetic resonance imaging (especially with T2-weighted, mapping or spectroscopy sequences). However, we have demonstrated in several studies [28–30] that low muscularity is associated with disease severity in SSc patients and, therefore, we suppose that reduced muscle mass is directly associated with limited exercise tolerance.

Further studies are needed to address the role of low muscle mass and complications related in SSc patients.

In conclusion, our study adds novel information regarding CWMA and exercise capacity in scleroderma patients without ILD.

CWMA represents an important variable and can be evaluated by the mediastinal window available in the HRCT images required for lung disease staging. Integrating CWMA in the evaluation of both ventilatory efficiency and exercise capacity can add a novel aspect and provide potential contributions to the clinicians for the global management in SSc patients.
